# A67 REDUCING INAPPROPRIATE GAMMA-GLUTAMYL TRANSFERASE TESTING FOR INPATIENTS: A QUALITY IMPROVEMENT INITIATIVE IN LAB WASTE REDUCTION APPLYING THE MODEL FOR CONTINUOUS IMPROVEMENT

**DOI:** 10.1093/jcag/gwad061.067

**Published:** 2024-02-14

**Authors:** T Afzaal, R AlRamdan, H Bualbanat, N C Howarth, G Malhi, D Hudson, I ChinYee, A Teriaky

**Affiliations:** Medicine, Western University, London, ON, Canada; University of Western Ontario Schulich School of Medicine & Dentistry, Western University Schulich School of Medicine & Dentistry, London, ON, CA, academic/medsch, London, ON, Canada; Gastroenterology, Western University, London, ON, Canada; Western University, London, ON, Canada; Gastroenterology, Western University, London, ON, Canada; Medicine, Western University, London, ON, Canada; Medicine, Western University, London, ON, Canada; Medicine, Western University, London, ON, Canada

## Abstract

**Background:**

Review of the literature identifies a rising trend in laboratory testing, with over 30% of tests estimated to be inappropriately repeated. Laboratory overutilization increases healthcare costs, and can lead to overdiagnosis, overtreatment and negative health outcomes. Indications for repeat Gamma Glutamyl Transferase (GGT) testing in adults are limited, particularly repeat testing within the same admission.

**Aims:**

Our aim was to reduce the inappropriate ordering of repeat GGT testing by 25% for all inpatients at the London Health Sciences Centre (LHSC) over a one-year study period.

**Methods:**

An interprofessional team was created to help engage relevant stakeholders, collect baseline data and reassess the indications for GGT testing. A combination of root cause analysis tools, specifically the Ishikawa diagram and Pareto chart, were employed to identify potential factors contributing to the overutilization of GGT testing. After prioritizing potential solutions, intervention bundles were developed, and Plan-Do-Study-Act (PDSA) cycles were created to target correctable factors. In PDSA cycle #1, the process started by eliminating GGT as a laboratory testing option in the three most commonly used admission order care sets. Considering the hierarchy of intervention effectiveness, PDSA cycle #2 involved implementing a computerized Clinical Decision Support (CDS) system to restrict the reordering of GGT tests within 72 hours of the same admission.

**Results:**

Baseline data showed that in 2022, a total of 62,542 GGT tests were ordered, with an average of approximately 5,200 GGT tests ordered per month. Of these, 16.4% were ordered through the top 3 most prevalent admission order care sets, and around 25% of all GGT tests were repeats within 72 hours of admission. Referring to Figure 1, PDSA cycle #1 yielded no significant reduction in GGT testing. PDSA cycle #2 successfully reduced the proportion of repeat GGT tests ordered by 12% within two months of implementation, leading to an estimated annualized cost savings of approximately $37,440.

**Conclusions:**

Our results establish the effectiveness of CDS systems in reducing laboratory testing overutilization, suggesting their superiority to individual care set targeting interventions, and emphasize the potential for cost-effective CDS development in contemporary healthcare.

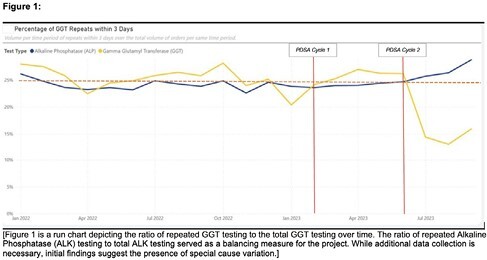

**Funding Agencies:**

None

